# Is IL-10 a Good Target to Inhibit Choroidal Neovascularisation in Age-Related Macular Disease?

**DOI:** 10.1371/journal.pmed.0030364

**Published:** 2006-08-15

**Authors:** Sue Lightman, Virginia Calder

## Abstract

Lightman discusses a new study that found that in a mouse model of choroidal neovascularisation (CNV), IL-10-deficient mice had decreased amounts of CNV.

Age-related macular disease (AMD) occurs most frequently in people aged 60 years and older and affects the central part of the retina, causing distortion or loss of central vision. The disease is the leading cause of blindness in people aged 60 years and older in the developed world and causes milder visual loss in a much greater number (about 25 percent of people aged 60 years and older in the developed world have some degree of visual loss because of AMD). AMD generally involves both eyes, although they may not be affected at the same time or to the same degree.

There are two forms of AMD: dry and wet. About 90 percent of patients with AMD have the dry atrophic type, for which there is currently no treatment, while 10 percent have wet AMD. Wet AMD causes the most severe visual loss and is the most aggressive form of the disease. In the wet form, choroidal blood vessels grow into the retina (choroidal neovascularisation [CNV]) but do not have the characteristic tight barriers of normal retinal blood vessels and so leak fluid into the retina. Such leakage often occurs under the central part of the retina—the macula—which is needed for visual detail. Scar tissue follows and leads to permanent severe visual loss.

In recent years, new forms of treatment for wet AMD have come into clinical practice following clinical trials. These new treatments are: (1) vitamins (the Age Related Eye Disease Study found that taking high levels of antioxidants and zinc can reduce the risk of developing advanced AMD by about 25 percent [1]); photodynamic therapy; and the most recently developed treatment, anti–vascular endothelial growth factor (anti-VEGF), which, when injected into the vitreous, has been helpful in preventing further visual loss and new vessel regression [2].

## A Mouse Model of CNV

In a study published in *PLoS Medicine*, Apte et al. examined the role of the macrophage component of the inflammatory response in CNV [3]. They used a mouse model in which CNV was induced in the eye with laser burns. In this mouse model, they looked at the possible role in CNV formation of the anti-inflammatory cytokine IL-10, which inhibits T cell and macrophage functions.

AMD is the leading cause of blindness in people aged 60 years and older in the developed world.

The researchers found that mice that were deficient in IL-10 (*IL-10* *^−/−^* mice) had increased inflammation and decreased amounts of CNV compared with wild-type mice. Systemic neutralisation of IL-10 in the wild-type mice produced a similar response; that is, neutralisation significantly reduced the laser-induced CNV in the wild-type mice. Intraocular injection of IL-10 in the *IL-10* *^−/−^* mice significantly increased the amount of neovascularisation to the level of the wild-type mice despite the reduced influx of macrophages.

The authors hypothesise that the effects of IL-10 in their animal model are most likely due to the increased inflammation seen in IL-10–deficient animals. They suggest that these results offer a novel approach to therapy in that IL-10 could be inhibited locally within the eye. Initially this treatment would be directed at patients with active CNV to limit or reverse vision loss, but, the authors suggest, it could be also used as a preventative therapy in patients who are at high risk of developing CNV.

## Current Treatments for CNV

CNV in the context of AMD is currently treated by laser therapy—either with an argon laser if the CNV is not sub-foveal, or with photodynamic therapy if the CNV is sub-foveal. More recently, anti-VEGF repeatedly injected directly into the vitreous cavity in the eye has been more successful in controlling CNV and is now used in clinical practice. Different types of anti-VEGF are currently being used: pegaptanib (anti-VEGF aptamer) is licensed for clinical use, and ranibizumab (anti-VEGF fragment) is about to be licensed. Bevacizumab, a full length anti-VEGF antibody, is rapidly becoming popular in clinical practice and has shown impressive outcomes, albeit in uncontrolled trials [2].

## Could the Anti-IL-10 Approach Be More Effective?

In Apte and colleagues' study [3], the researchers did not inject anti-IL-10 directly into the eye of the wild-type mouse to see if it had beneficial effects on CNV. Instead, the researchers inferred benefit from the experiments described above. Also they did not specifically study any preventative effects of anti-IL-10 upon CNV. Do their results provide data to support their ideas about the disease in humans? Is the biology of IL-10 in humans sufficiently understood, such that if we inhibit it the same predictable response will occur in all individuals in all situations?

In humans, corticosteroids have an impressive anti-inflammatory and anti-VEGF effect, and yet one of the ways they work is by up-regulating IL-10 production. Such up-regulation has also been shown to occur in the eye [4]. *IL-10* *^−/−^* mice are known to have higher corticosteroid levels in response to acute immune and physiologic stress [5] and are unlikely to have “normal” immunity. Therefore, Apte and colleagues need to repeat their experiments inducing CNV by laser treatment in wild-type mice and injecting anti-IL-10 into the eye to see if it reduces CNV.

In experimental autoimmune encephalomyelitis, IL-10 is thought to be the major cytokine for recovery. The source of IL-10 includes B cells thought to be from the peripheral lymphoid organs and from CD4^+^ CD25^+^ T cells [6], and yet in Apte and colleagues' study the researchers looked at only macrophages. IL-10 is produced by a variety of cell types but also is thought to be inhibitory for most cells and signalling pathways, with the exception of myofibroblasts [7]. However, in a skin-culture system IL-10 was found to down-regulate collagen synthesis [8], so if anti-IL-10 were given when active neovascularisation was occurring, healing, closure of the new vessels, and scarring could be increased, causing more of a problem.

## Further Unanswered Questions

Patients may vary in the amount of inflammation associated with CNV. Does a patient aged 55 years, for example, have a different inflammatory response from a patient aged 80 years? Are variations in the cellular mix in the choroid and retina going to vary the effect of giving anti-IL-10? Compounding this, patients have different balances of cytokines, and some IL-10 cytokine gene polymorphisms vary the amount of IL-10 produced to any given stimulus [9]. This may mean that in some patients the amount of IL-10 produced locally is low and in others it is high, which will also apply to other cytokines in the inflammatory area.

Other cell types and cytokines present, and the balance of cytokines, may vary between individuals, yet it is not possible to know this by clinical examination of the patient. In terms of preventing visual loss, does short-term administration of anti-IL-10 predict the effects of long-term administration? Does acute disease induction give the same phenotype of the chronic disease process? Would it have the same effect if given before CNV has occurred, when the cellular mix in the choroid and retina is likely to be different? Injecting anti-cytokines into the eye to prevent disease is an interesting approach, but Apte and colleagues' study does not provide any data to show that it might be useful in prevention of CNV, and the question of long-term toxicity to the retina is paramount.

## Conclusion

Apte and colleagues' preliminary findings are intriguing, but they are too early to have direct relevance to treating humans. In asthma, studies in mice indicated that IL-5 was a key cytokine, but its inhibition had no effect on bronchiolar constriction in humans [10]. Animal models may help in understanding the interactions of the components of a given disease response, but the situation can be completely different in humans.

## Abbreviations

AMD: age-related macular disease
CNV: choroidal neovascularisation
VEGF: vascular endothelial growth factor
